# Integration of digital technologies in orthodontic treatment of children and adolescents: a prospective non-randomized comparative study on personalized therapy in the 12–18 age group

**DOI:** 10.3389/fmed.2026.1853221

**Published:** 2026-07-01

**Authors:** Jingying Li, Yucheng Gao, Huaan Xia, Andrey Mikhailovich Dybov, Olesya Viktorovna Dudnik

**Affiliations:** Department of Orthodontics and Prevention of Dental Diseases, E.V. Borovsky Institute of Dentistry, Sechenov First Moscow State Medical University, Moscow, Russia

**Keywords:** adolescents, clear aligners, clinical efficiency, digital orthodontics, fixed appliances, patient-reported outcomes, remote monitoring

## Abstract

**Background and objectives:**

Clear aligner therapy combined with digital planning and remote monitoring is increasingly adopted in orthodontics, yet prospective comparative data in adolescents remain limited. This prospective non-randomized study compared short-term outcomes of an aligner-based digital orthodontic pathway versus conventional fixed appliances in adolescents with mild-to-moderate mandibular crowding.

**Materials and methods:**

In this prospective non-randomized comparative study, 78 adolescents (aged 12–18 years) with mild-to-moderate mandibular anterior crowding received either conventional fixed appliances or a digital pathway incorporating intraoral scanning, virtual treatment planning, clear aligners, and AI-assisted remote monitoring. Follow-up lasted 24 weeks. The primary outcome was change in Little’s Irregularity Index (LII). Secondary outcomes included treatment duration, clinic visits, chair time, patient satisfaction, and caregiver satisfaction.

**Results:**

Both groups showed substantial alignment improvement. Mean LII reduction was 5.09 ± 0.57 mm in the digital group versus 4.29 ± 0.37 mm in the conventional group, with the between-group difference of 0.80 mm (95% CI, 0.59–1.02 mm), not reaching the prespecified clinical relevance threshold of 1.2 mm. The digital pathway required fewer in-office visits (2.46 vs. 4.46, *p* < 0.001), shorter chair time (93.1 vs. 170.8 min, *p* < 0.001), and shorter alignment duration (21.4 vs. 24.0 weeks, *p* < 0.001). Patient satisfaction (8.85 vs. 6.97, *p* < 0.001) and caregiver satisfaction (92.3% vs. 69.2%, *p* = 0.019) favored the digital pathway. Unplanned visits were numerically less frequent in the digital group (2.6% vs. 15.4%).

**Conclusion:**

In this non-randomized study, the digital orthodontic pathway and conventional fixed appliances produced comparable short-term alignment outcomes in adolescents. The digital approach demonstrated advantages in treatment efficiency, resource utilization, and patient experience. These findings support the integration of digital technologies in adolescent orthodontic care, though randomized trials are needed to confirm these observations.

## 1 Introduction

Orthodontic treatment in adolescents aims not only to improve dental alignment and occlusal function, but also to support oral health and treatment acceptance during a period in which cooperation may vary. Conventional fixed appliances remain widely used and effective; however, they require regular in-person follow-up, chairside adjustments, and close supervision of oral hygiene and appliance integrity. In adolescent patients, these demands may create additional challenges for both families and clinicians (1).

Digital orthodontics has transformed clinical practice over the past decade. Technologies like intraoral scanning and 3D treatment planning now enable precise virtual setups, while remote monitoring allows clinicians to track patient progress through smartphone-submitted images (2–9). These innovations promise to reduce in-person appointments while maintaining treatment oversight. For adolescents facing school schedules and social concerns, such tools may be particularly valuable for maintaining treatment engagement (10, 11).

Remote monitoring systems supported by artificial intelligence can analyze patient-submitted intraoral images, identify tracking problems, and alert clinicians to possible deviations from the planned treatment sequence (3, 6, 8, 12). In clear aligner therapy, such systems may help detect poor fit, delayed tooth movement, or inconsistent wear earlier than routine office visits would allow (8, 12). At the same time, digital workflows may reduce the burden of repeated appointments and improve treatment transparency for patients and parents (8, 10, 12, 13).

While digital orthodontics adoption accelerates, high-quality prospective comparative studies in adolescent populations remain scarce (12, 14–20). Previous studies have examined individual components of digital orthodontics, including aligner effectiveness, digital planning, and remote monitoring, but few have compared short-term clinical outcomes together with patient-reported experience and healthcare utilization (8, 10, 12, 18–20).

**FIGURE 1:**
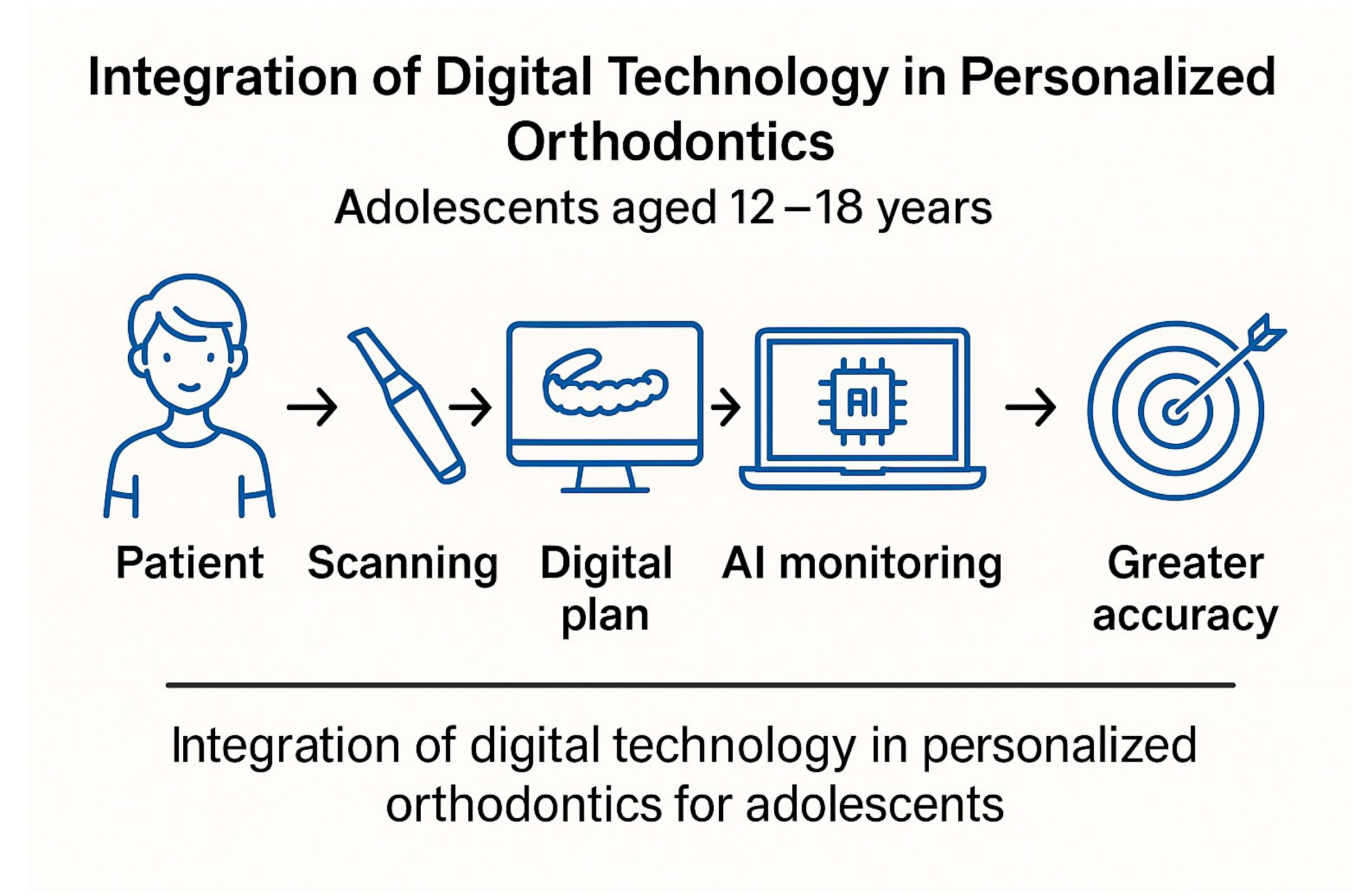
Integration of digital technology in personalized orthodontics.

The aim of the present study was to compare a conventional fixed-appliance pathway with an aligner-based digital orthodontic pathway combining intraoral scanning, virtual treatment planning, clear aligners, and remote monitoring in adolescents with mild-to-moderate mandibular crowding under routine prospective clinical conditions. The primary objective was to compare short-term alignment improvement. The secondary objectives were to evaluate treatment efficiency, clinic resource use, and patient- and caregiver-reported outcomes over a 24-week observation period. The digital treatment pathway evaluated in this study is summarized in Figure 1.

A schematic illustration of the digital orthodontic pathway used in the study, including patient enrollment, intraoral scanning, virtual treatment planning, clear aligner fabrication, AI-assisted remote monitoring, and clinical follow-up.

## 2 Materials and methods

### 2.1 Study design and setting

**FIGURE 2:**
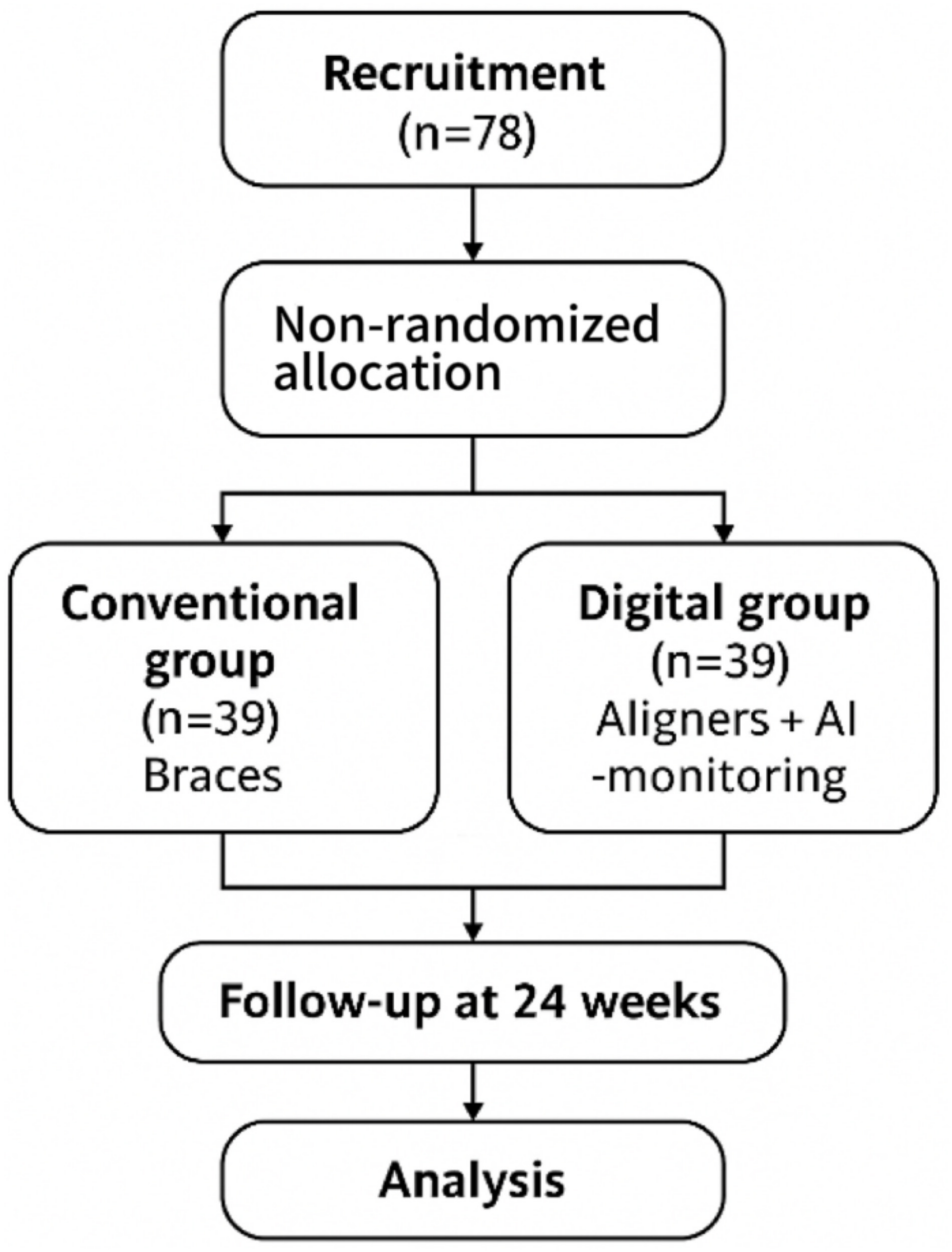
Study flow diagram.

The overall study design and participant flow are shown in Figure 2.

Flowchart showing participant recruitment, eligibility assessment, allocation to the conventional or digital treatment group, and completion of the 24-week follow-up period.

This was a prospective, non-randomized, two-arm parallel-group comparative study conducted from November 2024 to January 2026 at three orthodontic centers in a metropolitan area. Each participant was followed for 24 weeks during the initial leveling and alignment stage of treatment. The study compared short-term outcomes between a conventional fixed-appliance pathway and an aligner-based digital orthodontic pathway with remote monitoring.

All procedures were conducted in accordance with the Declaration of Helsinki. The study protocol was approved by the Institutional Review Board of Sechenov First Moscow State Medical University (Approval No. 26–24; approval date: 24 October 2024). Written informed consent was obtained from all participants and their legal guardians before enrollment.

### 2.2 Participants

A total of 78 adolescents aged 12–18 years were enrolled. Eligibility criteria included Class I or mild Class II malocclusion, mild-to-moderate mandibular anterior crowding defined as Little’s Irregularity Index between 4 and 9 mm, satisfactory oral hygiene, and no previous orthodontic treatment. Exclusion criteria were craniofacial anomalies, active periodontal disease, relevant systemic illness, and treatment plans involving orthognathic surgery.

Participants were allocated to either the conventional group or the digital group under routine clinical conditions through clinician- and caregiver-informed decision-making, with an effort to maintain approximate balance in age and sex between groups. Because treatment allocation was not randomized, the study was designed to evaluate comparative pathway-level outcomes rather than isolate the independent effect of any single technological component.

### 2.3 Intervention protocol

Participants in the conventional group received treatment with fixed metal appliances according to routine clinical practice. The protocol included alginate impressions, manual bracket placement, archwire sequencing, and scheduled in-person follow-up every 4–5 weeks. Adjustments were made at chairside based on routine clinical examination.

Participants in the digital group were treated using a digital orthodontic workflow that included intraoral scanning (iTero Element; Align Technology, San Jose, CA, United States), virtual treatment setup (ClinCheck Pro; Align Technology), customized clear aligners (Invisalign; Align Technology), and remote monitoring via the DentalMonitoring platform (DentalMonitoring, Paris, France). Patients submitted weekly intraoral images through a smartphone-based application. These images were assessed by the platform’s analytical system to identify tracking issues, possible fit discrepancies, and adherence-related concerns. When indicated, clinicians provided remote feedback or arranged in-person assessment.

All treatments in both groups were performed by board-certified orthodontists with at least 5 years of clinical experience. All participants received standardized oral hygiene instructions and routine motivational guidance.

### 2.4 Outcomes

The primary outcome was the change in Little’s Irregularity Index (LII) from baseline to 24 weeks.

Secondary outcomes included the duration of the initial alignment phase, number of in-office visits during follow-up, cumulative chair time, deviation between planned and achieved tooth movement at 24 weeks, patient satisfaction, caregiver satisfaction, appliance-specific adherence, and unplanned or emergency visits.

Baseline data included age, sex, oral hygiene index (OHI-S), baseline Little’s Irregularity Index, and baseline satisfaction measured on a 10-point visual analog scale (VAS).

### 2.5 Outcomes assessment

Little’s Irregularity Index was recorded in millimeters at baseline and at 24 weeks. Alignment duration was defined as the number of weeks required to complete the initial leveling and alignment phase. The number of in-office visits and total chair time were obtained from clinical records.

Deviation between planned and achieved tooth movement was assessed at 24 weeks by comparing the achieved mandibular anterior tooth positions with the predefined treatment objectives established at baseline for each participant. In the digital group, planned positions were derived from the virtual treatment setup generated in ClinCheck Pro (Align Technology, San Jose, CA, United States). In the conventional group, planned positions were defined from the baseline orthodontic treatment plan established by the treating orthodontist on the basis of pretreatment records, including clinical examination, study models or digital scans, and standard alignment objectives for the mandibular anterior segment. Digital models obtained at baseline and at 24 weeks were superimposed and analyzed using 3Shape Ortho Analyzer (3Shape A/S, Copenhagen, Denmark). The linear discrepancy between planned and achieved positions was measured in millimeters.

All measurements were performed by a single calibrated examiner. Examiner blinding to group allocation was not feasible because treatment records and appliance systems differed visibly between groups. To reduce random measurement error, a randomly selected subset of cases was remeasured after a 1-week interval under the same conditions. Intra-examiner reliability was assessed using intraclass correlation coefficient (ICC), with values above 0.90 indicating excellent reliability.

Specifically, intra-examiner reliability was assessed in a randomly selected subset of 15 participants, including cases from both treatment groups. The same calibrated examiner repeated Little’s Irregularity Index (LII) and planned–achieved movement deviation measurements after a 1-week interval without reference to the first measurements. Intraclass correlation coefficients (ICCs) were calculated to evaluate reproducibility.

Patient satisfaction was measured at 24 weeks using a 10-point visual analog scale. Appliance-specific adherence was assessed separately in each group. In the digital group, adherence was based on remote monitoring records, with high adherence defined as greater than 90% prescribed wear time. In the conventional group, adherence was assessed on the basis of appointment attendance and appliance integrity, including bracket and wire failures. Because these adherence measures were not directly equivalent across appliance systems, they were interpreted as appliance-specific indicators rather than interchangeable behavioral metrics.

Caregiver satisfaction was recorded at the 24-week assessment using a structured end-of-follow-up questionnaire. Unplanned visits were defined as unscheduled emergency appointments related to appliance problems, discomfort, or treatment tracking concerns.

### 2.6 Sample size calculation

An a priori power analysis indicated that 34 participants per group would provide 80% power to detect a minimum clinically meaningful between-group difference of 1.2 mm in Little’s Irregularity Index at a two-sided alpha level of 0.05, assuming a standard deviation of 1.5 mm. The 1.2-mm threshold was defined a priori as the minimum between-group difference considered clinically relevant for short-term LII reduction. This value was selected based on the millimeter-based nature of LII scoring, expected measurement variability, and clinical judgment that smaller differences would be unlikely to alter treatment decision-making during the initial alignment phase. To allow for potential attrition, the target sample size was increased to 78 participants.

### 2.7 Statistical analysis

Statistical analyses were performed using IBM SPSS Statistics version 26.0. Data distribution was assessed using the Shapiro–Wilk test. Continuous variables are presented as mean ± standard deviation and were compared using the independent-samples *t*-test or Mann–Whitney U-test, as appropriate. Within-group changes over time were assessed using paired tests appropriate to data distribution. Categorical variables were compared using Pearson’s chi-squared test or Fisher’s exact test, as appropriate. Spearman’s rank correlation was used to explore associations between adherence, treatment predictability, and satisfaction.

A *p* < 0.05 was considered statistically significant. Mean differences and 95% confidence intervals are reported where available. Because the study was powered for the primary outcome, secondary outcome analyses were interpreted as supportive comparative findings. Baseline characteristics of the study population are presented in Table 1.

**TABLE 1:** Baseline characteristics of participants.

Variable	Conventional group (*n* = 39)	Digital group (*n* = 39)	*p*-value
Number of participants	39	39	–
Mean age (years)	14.72 ± 1.49	14.72 ± 1.49	1.000
Sex (female/male)	21 / 18	22 / 17	1.000
Baseline Little’s Index (mm)	6.48 ± 0.97	6.46 ± 0.93	0.915
Mean oral hygiene status (OHI-S)	1.83 ± 0.30	1.81 ± 0.27	0.722
Previous orthodontic treatment (%)	0%	0%	–
Baseline satisfaction score (VAS 0–10)	5.74 ± 1.02	5.74 ± 1.02	1.000

## 3 Results

### 3.1 Baseline characteristics

Seventy-eight participants were included in the analysis, with 39 adolescents in each group. Intra-examiner reliability was excellent, with ICCs greater than 0.99 for all repeated measurements (baseline LII, post-treatment LII, and deviation from planned tooth movement). Baseline characteristics were similar between the two groups. The mean age was 14.72 ± 1.49 years in both the conventional group and digital group (*p* = 1.000). The sex distribution was similar between groups (22 females and 17 males in the digital group, and 21 females and 18 males in the conventional group). Baseline Little’s Irregularity Index was 6.48 ± 0.97 mm in the conventional group and 6.46 ± 0.93 mm in the digital group (*p* = 0.915), and the mean oral hygiene index was 1.8 ± 0.4 in each group. Baseline satisfaction scores were 5.74 ± 1.02 for the conventional group and 5.74 ± 1.02 for the digital group (*p* = 1.000).

### 3.2 Primary outcome: alignment improvement

Both groups showed marked improvement in mandibular anterior alignment over the 24-week observation period. In the digital group, Little’s Irregularity Index decreased from 6.46 ± 0.93 mm to 1.37 ± 0.41 mm, corresponding to a mean reduction of 5.09 mm. In the conventional group, the index decreased from 6.48 ± 0.97 mm to 2.20 ± 0.60 mm, corresponding to a mean reduction of 4.29 mm.

**FIGURE 3:**
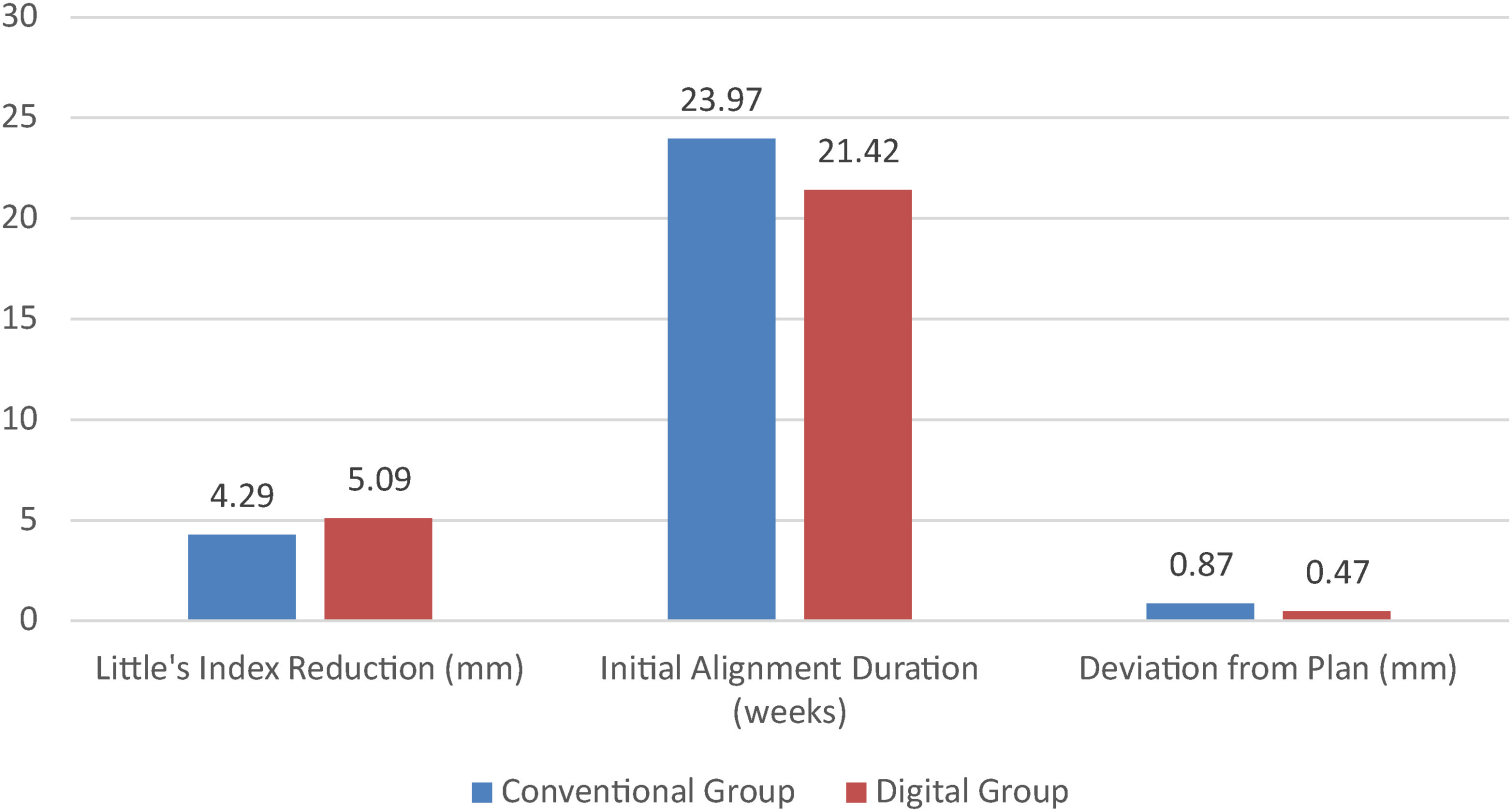
Comparison of clinical outcomes between treatment groups.

The primary between-group comparison focused on the magnitude of change in Little’s Irregularity Index from baseline to 24 weeks. The digital group showed a numerically greater reduction than the conventional group; however, the observed between-group mean difference was 0.80 mm (95% CI, 0.59–1.02 mm), which remained entirely below the prespecified threshold for clinical relevance (1.2 mm). Accordingly, the short-term alignment improvement achieved by the two treatment pathways was interpreted as broadly comparable from a clinical perspective. A graphical summary of the main clinical outcomes is provided in Figure 3.

Bar chart comparing major clinical outcomes, including reduction in Little’s Irregularity Index, duration of initial alignment, and deviation from planned tooth movement in the conventional and digital groups.

### 3.3 Treatment efficiency and resource use

The mean duration of the initial alignment phase was shorter in the digital group than in the conventional group (21.42 ± 1.37 weeks vs. 23.97 ± 2.09 weeks; *p* < 0.001). The digital group also required fewer in-office visits during follow-up (2.46 ± 0.68 vs. 4.46 ± 1.05; *p* < 0.001). Total chair time was lower in the digital group (93.08 ± 13.89 min) than in the conventional group (170.77 ± 25.33 min; *p* < 0.001).

A higher proportion of patients in the digital group completed the observation period with two or fewer in-office visits (64.1% vs. 7.7%; *p* < 0.001). Unplanned or emergency visits were numerically less frequent in the digital group than in the conventional group (1/39, 2.6% vs. 6/39, 15.4%), although this difference did not reach statistical significance using Fisher’s exact test (*p* = 0.108).

### 3.4 Treatment predictability

**FIGURE 4:**
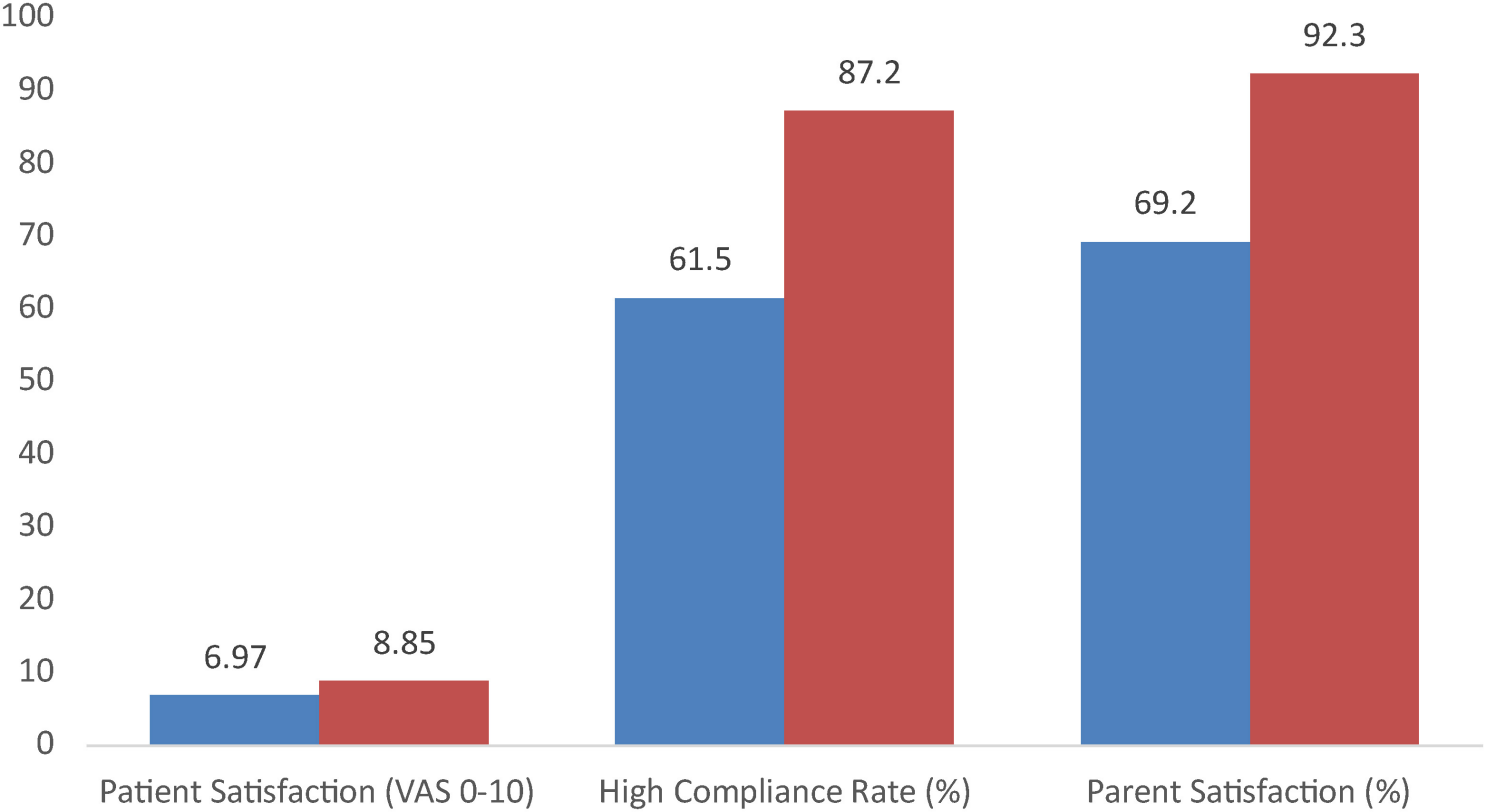
Patient and caregiver satisfaction metrics.

Deviation between planned and achieved tooth movement at 24 weeks was smaller in the digital group than in the conventional group (0.47 ± 0.15 mm vs. 0.87 ± 0.27 mm; mean difference –0.40 mm; 95% CI, –0.50 to –0.30 mm; *p* < 0.001). This finding suggests closer agreement between planned and achieved tooth positions within the digital treatment pathway. However, this outcome should be interpreted cautiously because the treatment systems and planning frameworks differed between groups, and the metric should therefore be regarded as a pathway-level comparative indicator rather than a strictly equivalent cross-system measure. Patient-reported and caregiver-reported outcomes are summarized in Figure 4.

Comparative summary of patient satisfaction, appliance-specific adherence, and caregiver satisfaction in the conventional and digital groups.

### 3.5 Patient-reported and caregiver-reported outcomes

Patient satisfaction at 24 weeks was higher in the digital group than in the conventional group (8.85 ± 0.78 vs. 6.97 ± 0.87; *p* < 0.001). Caregiver satisfaction was also more favorable in the digital group, with 92.3% of caregivers rating the treatment process as very satisfactory compared with 69.2% in the conventional group (Fisher’s *p* = 0.019).

Appliance-specific adherence indicators were descriptively more favorable in the digital pathway. High adherence was recorded in 34/39 patients in the digital group and in 24/39 patients in the conventional group. However, because adherence was operationalized differently across appliance systems, this comparison was considered exploratory (Fisher’s *p* = 0.018) and was not interpreted as a direct measure of equivalent compliance.

### 3.6 Remote monitoring activity

**FIGURE 5:**
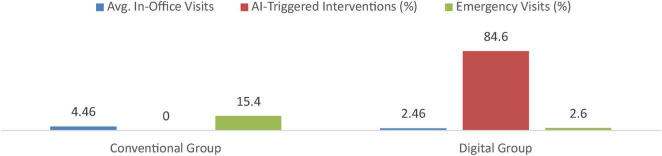
Remote monitoring and efficiency metrics.

In the digital group, weekly image submissions allowed continuous review of treatment progress. A total of 84.6% of patients triggered at least one remote monitoring alert or intervention during the study period. These contacts most often involved fit assessment, tracking evaluation, or adherence-related feedback. No comparable remote monitoring mechanism was available in the conventional group. Key efficiency indicators related to remote monitoring are illustrated in Figure 5. Detailed clinical outcomes at 24 weeks are presented in Table 2. Patient satisfaction and adherence-related findings are summarized in Table 3. Remote monitoring and clinical efficiency metrics are presented in Table 4.

**TABLE 2:** Clinical outcomes at 24 Weeks.

Outcome measure	Conventional group (*n* = 39)	Digital group (*n* = 39)	Mean difference (digital - conv)	95% CI	*p*-value
Post-treatment Little’s Index (mm)	2.20 ± 0.60	1.37 ± 0.41	–0.83	–1.06 to –0.59	< 0.001
Mean reduction in Little’s Index (mm)	4.29 ± 0.37	5.09 ± 0.57	0.80	0.59 to 1.02	< 0.001
Treatment duration (weeks)	23.97 ± 2.09	21.42 ± 1.37	–2.56	-3.36 to –1.76	< 0.001
Number of in-office visits	4.46 ± 1.05	2.46 ± 0.68	–2.00	–2.40 to –1.60	< 0.001
Deviation from planned tooth movement (mm)	0.87 ± 0.27	0.47 ± 0.15	–0.40	–0.50 to –0.30	< 0.001

**TABLE 3:** Patient satisfaction and appliance-specific adherence indicators at 24 weeks.

Metric	Conventional group (*n* = 39)	Digital group (*n* = 39)	*p*-value/Interpretation
Mean satisfaction score (VAS, 0–10)	6.97 ± 0.87	8.85 ± 0.78	< 0.001
High appliance-specific adherence rate (% of patients)	24/39 (61.5%)	34/39 (87.2%)	Exploratory (Fisher’s *p* = 0.018)
Caregiver satisfaction (rated “very satisfactory”)	27/39 (69.2%)	36/39 (92.3%)	Fisher’s *p* = 0.019

Comparison of adherence rates is strictly exploratory due to different cross-system tracking modalities.

**TABLE 4:** Remote monitoring and clinical efficiency.

Metric	Conventional group (*n* = 39)	Digital group (*n* = 39)	*p*-value/Interpretation
Mean number of in-office visits	4.46 ± 1.05	2.46 ± 0.68	<0.001
Patients completing treatment with ≤ 2 visits	3/39 (7.7%)	25/39 (64.1%)	Fisher’s *p* < 0.001
Average total chair time (minutes)	170.77 ± 25.33	93.08 ± 13.89	<0.001
Patients with remote monitoring alerts	0/39 (0%)	33/39 (84.6%)	Not Applicable
Unplanned/emergency visits	6/39 (15.4%)	1/39 (2.6%)	Fisher’s *p* = 0.108

Fisher’s exact test was applied for categorical variables with small cell counts (unplanned/emergency visits).

Graphical comparison of the frequency of in-office visits, remote interventions, total chair time, and unplanned visits between the conventional and digital pathways.

## 4 Discussion

Our 24-week study found that adolescents with mild-to-moderate mandibular crowding achieved substantial short-term alignment improvement with both the aligner-based digital orthodontic pathway and conventional fixed appliances. The mean between-group difference in Little’s Irregularity Index reduction was 0.80 mm, and its 95% confidence interval remained below the prespecified 1.2-mm threshold for clinical relevance. Therefore, although the digital pathway showed a statistically greater LII reduction, the magnitude of this difference should be interpreted as clinically limited rather than as evidence of a decisive superiority in tooth-alignment capacity.

Clear differences emerged in treatment logistics. Patients in the digital group attended fewer in-office visits, spent less cumulative time in the dental chair, and completed the initial alignment phase sooner. These findings are consistent with the growing literature suggesting that remote orthodontic monitoring may reduce the burden of scheduled office visits. A recent systematic review by Sangalli et al. reported that Dental Monitoring added to standard orthodontic care significantly decreased the number of in-office visits and showed a possible trend toward improved aligner fit, although the authors emphasized the low quality and heterogeneity of the available evidence (21). Similarly, Lam et al., in a single-center randomized controlled trial of clear aligner patients, found that Dental Monitoring resulted in fewer clinical appointments, but it did not improve all efficiency-related or patient-experience outcomes (22). These studies support our observation of fewer in-office visits in the digital pathway, but they also reinforce the need for cautious interpretation.

The interpretation of alignment outcomes should also be considered in light of recent evidence on clear aligner therapy. Contemporary reviews indicate that clear aligners can provide clinically acceptable outcomes in selected mild-to-moderate malocclusion cases, while predictability may vary according to case complexity, type of tooth movement, patient cooperation, and treatment protocol (23). This context is consistent with the present finding that both pathways produced substantial alignment improvement, whereas the absolute between-group difference in LII reduction remained below the prespecified threshold for clinical relevance.

In adolescent care, where school attendance, caregiver availability, and travel requirements may influence continuity of care, reductions in visit burden and chair time may be clinically meaningful even when primary alignment outcomes are broadly comparable. Therefore, the main observed advantages of the digital pathway in this study were related to clinical logistics and patient experience rather than a clinically decisive improvement in tooth-alignment magnitude.

Patient satisfaction was higher in the digital group, and caregiver responses followed the same general pattern. Several factors may have contributed to this finding, including the reduced number of office visits, the esthetic acceptability of aligners, and the perception of continuous supervision created by remote monitoring. However, the present study was not designed to separate the relative contribution of appliance type, digital communication, and visit reduction to overall satisfaction.

The satisfaction-related findings should also be interpreted in the broader context of tele-orthodontics and digitally supported patient communication. A recent survey of American Association of Orthodontists members reported that tele-orthodontics is commonly perceived as useful for aligner monitoring, patient communication, education, and reduction of unnecessary visits, but also identified concerns regarding privacy, patient safety, diagnostic limitations, and informed consent (24). These considerations are particularly relevant in adolescent orthodontics, where caregiver involvement, patient motivation, and familiarity with smartphone-based tools may influence both satisfaction and adherence-related outcomes.

Deviation between planned and achieved tooth movement was smaller in the digital group. This observation may reflect closer monitoring and tighter correspondence between virtual planning and clinical execution within the digital pathway. At the same time, this result should be interpreted with caution because treatment predictability was assessed across two fundamentally different treatment systems with different planning frameworks. It is therefore more appropriate to interpret this outcome as a comparative pathway-level indicator than as a strictly equivalent cross-system measure.

At the same time, AI-assisted remote monitoring should not be interpreted as an error-free or independently validated causal mechanism. Ferlito et al. prospectively assessed artificial intelligence-based remote monitoring in clear aligner therapy and reported concerns regarding the consistency of repeated GO/NO-GO instructions and the detection of tracking issues (25). This evidence supports a balanced interpretation of the present findings: remote monitoring may contribute to closer surveillance and earlier identification of fit or tracking concerns, but it should complement rather than replace clinical judgment.

Several limitations should be considered when interpreting the present findings. First, the study used a prospective non-randomized design, and treatment allocation was determined under routine clinical conditions rather than by random assignment. Accordingly, selection bias and residual confounding cannot be excluded. Families who selected the digital aligner pathway may have differed systematically from those treated with conventional fixed appliances in terms of socioeconomic status, esthetic expectations, baseline motivation, familiarity with smartphone-based tools, and parental involvement. These factors may have influenced treatment efficiency, appliance-specific adherence indicators, and satisfaction outcomes.

Second, the interventions differed simultaneously at multiple levels, including appliance type, treatment planning method, and monitoring strategy. As a result, the observed differences cannot be attributed specifically to remote monitoring or artificial intelligence alone. The present findings should therefore be interpreted as comparative observations on two clinical pathways rather than as evidence for the isolated effect of any single digital component.

Third, outcome assessment was performed by a single non-blinded examiner. Although intra-examiner reliability was excellent in the repeated-measurement subset, examiner blinding was not feasible because treatment records and appliance systems differed visibly between groups. Therefore, potential measurement bias cannot be fully excluded, particularly for LII and planned–achieved movement deviation outcomes.

A further limitation concerns adherence assessment. In the digital group, adherence was derived from prescribed aligner wear behavior documented through remote monitoring records. In the conventional group, adherence reflected appointment attendance and appliance integrity. These constructs are clinically relevant within each treatment arm, but they are not directly equivalent and should not be interpreted as interchangeable behavioral metrics. For this reason, adherence findings were treated as appliance-specific exploratory indicators.

Additional limitations include the relatively short follow-up period, which did not permit assessment of long-term stability or biologic sequelae such as root resorption; the restriction of the sample to adolescents with mild-to-moderate crowding; and the absence of prospective registration in a public clinical trial registry. This study should therefore be interpreted as hypothesis-generating comparative clinical evidence rather than confirmatory trial-level evidence.

Future studies would be strengthened by randomized allocation, standardized adherence measures, blinded or independently replicated outcome assessment, longer follow-up, and designs that compare similar appliance systems with and without remote monitoring. Such approaches would allow a more direct estimate of the independent contribution of remote digital supervision to clinical efficiency, predictability, and patient experience.

## 5 Conclusion

In this prospective non-randomized comparative study, an aligner-based digital orthodontic pathway and conventional fixed appliances produced broadly comparable short-term improvement in mandibular anterior alignment in adolescents with mild-to-moderate crowding. The digital pathway demonstrated advantages in reducing in-office visits, chair time, and treatment duration, while achieving higher patient and caregiver satisfaction. Because treatment allocation was not randomized and appliance type, planning approach, and monitoring strategy differed simultaneously between groups, these findings should be interpreted as pathway-level comparative observations rather than evidence of an isolated benefit of artificial intelligence or remote monitoring alone. Randomized controlled trials with longer follow-up are needed to confirm these observations and assess long-term outcomes.

## Data Availability

The original contributions presented in this study are included in the article/supplementary material, further inquiries can be directed to the corresponding author.
